# The preventive effect of the impaired liver function for antiemetic therapy against chemotherapy-induced nausea and vomiting in hepatocellular carcinoma patients

**DOI:** 10.3164/jcbn.17-57

**Published:** 2017-10-19

**Authors:** Tomohiro Nishikawa, Akira Asai, Norio Okamoto, Hidetaka Yasuoka, Ken Nakamura, Keisuke Yokohama, Hideko Ohama, Yusuke Tsuchimoto, Shinya Fukunishi, Yasuhiro Tsuda, Kazuhiro Yamamoto, Kazuhide Higuchi

**Affiliations:** 1 2nd Department of Internal Medicine, Osaka Medical College, 2-7 Daigakumachi, Takatsuki, Osaka 569-8686, Japan; 2 Medical Laboratory, Osaka Medical College, 2-7 Daigakumachi, Takatsuki, Osaka 569-8686, Japan; 3 Faculty of Nursing, Osaka Medical College, 2-7 Daigakumachi, Takatsuki, Osaka 569-8686, Japan; 4 Faculty of Radiology, Osaka Medical College, 2-7 Daigakumachi, Takatsuki, Osaka 569-8686, Japan

**Keywords:** HCC, TACE, HAIC, CINV, NK_1_ antagonist

## Abstract

Transarterial chemoembolization and hepatic arterial infusion chemotherapy are recommended for the treatment in patients with intermediate stage of hepatocellular carcinoma. Impaired liver function was sometime observed in patients with hepatocellular carcinoma after transarterial chemoembolization or hepatic arterial infusion chemotherapy. However, what kinds of factors deeply influence in impaired liver function are not clear. A retrospective study was performed to evaluate the risk factors of impaired liver function in cisplatin-naïve patients treated with these therapies using cisplatin. Prior to and 2 months after these therapies, we analyzed the liver function by Child-Pugh score in these patients. For assessing the severity of chemotherapy-induced nausea and vomiting, we utilized the Common Terminology Criteria for Adverse Events ver. 4.0. In hepatocellular carcinoma patients received these therapies using cisplatin, the cancer stage and treatment without neurokinin-1 (NK_1_) antagonist were found to be independent risk factors of the impaired liver function. The treatment with NK_1_ antagonist was effective in reducing chemotherapy-induced nausea and vomiting and patients treated with NK_1_ antagonist kept their liver functions after cisplatin-used these therapies. The treatment with NK_1_ antagonist was effective in chemotherapy-induced nausea and vomiting and prevented the impaired liver function associated with cisplatin-used these therapies in hepatocellular carcinoma patients.

## Introduction

Hepatocellular carcinoma (HCC) is the fifth most common cancer, and major clinical risk factors for HCC include infection with hepatitis B virus (HBV) or hepatis C virus (HCV), alcoholic liver disease, non-alcoholic fatty liver disease, and oxidative stress.^(1)^ Most of these risk factors cause inflammation of the liver and lead to cirrhosis, which is present in 80 to 90% of patients with HCC.^(2)^ The prognosis of patients with HCC depends on both residual liver function and tumor burden.^(3)^ There are several approaches to the treatment of HCC, and the selection of treatment is driven by residual liver function, the cancer stage and resources available.^(4,5)^ The Barcelona Clinic Liver Cancer (BCLC) staging is a useful assessment tool that incorporates data on the liver function as determined by the Child-Pugh classification system, number and size of nodules, cancer symptoms, and patient’s performance status.^(6)^ Transarterial chemoembolization (TACE) and hepatic arterial infusion chemotherapy (HAIC) are recommended for HCC patients with intermediate-stage BCLC and improve the 2-year survival rate compared with that of more conservative therapy. TACE or HAIC increase the risk for ischemic necrosis of the non-tumoral liver, and this adverse event may lead to severe liver failure. Therefore, maintaining liver function is important for patients receiving these treatments.^(7–9)^

Chemotherapy-induced nausea and vomiting (CINV) have been reported as the most distressing adverse side effects in more than 90% of the patients treated with highly emetogenic antitumor agents, especially cisplatin.^(10,11)^ After the 1990s, cisplatin, based on guidance for the prevention of CINV, has been used for TACE or HAIC.^(12)^ CINV sometimes leads the malnutrition in patients.^(10)^ The malnutrition is very common in patients with advanced hepatic disease and gets worse with the severity of liver dysfunction because of inadequate and/or quality oral intake, maldigestion, malabsorption, increased energy expenditure and altered substrate demands.^(13–15)^ And the malnutrition which leads impaired liver function, is an independent risk factor for increased morbidity and mortality in these patients.^(16)^ Therefore, the treatment for the CINV which leads the malnutrition, is very important for the patients with HCC after TACE or HAIC.

CINV is differentiated into two categories: acute CINV (mostly serotonin related), occurring within 24 h of initial administration of the chemotherapy; delayed CINV (in part substance P related), occurring 24 h to several days after the initial treatment.^(17)^ Introduction of serotonin (5-hydroxytryptamine-3) receptor antagonist (5-HT_3_ antagonist) in the early 1990s represented a major advance in the management of acute CINV.^(18)^ Substance P is a regulatory peptide found in areas of the central nerve system and in the gastrointestinal tract and is believed to be an essential component of the emetic reflex. The actions of substance P are mediated through the neurokinin-1 (NK_1_) receptor.^(19)^ Selective antagonist of the NK_1_ receptor have demonstrated antiemetic activity against central, peripheral, and combined emetic stimuli.^(20)^ The combination therapy with NK_1_ antagonist reduces the CINV associated with the highly emetogenic cisplatin-based chemotherapy according to the American Society of Clinical Oncology Clinical Practice Guideline.^(21)^ However, what kinds of factors deeply influence in impaired liver function and the effect of NK_1_ antagonist against CINV on the impaired liver function is unclear in HCC patients who are treated with either TACE or HAIC. The present study is the first report on the effect on liver function of the antiemetic therapy in HCC patients treated with TACE or HAIC.

## Patients and Methods

### Subjects

From 2008 to 2015, 141 cisplatin-naïve patients with HCC who received TACE or HAIC treatment using cisplatin were enrolled at Osaka Medical College. 4 patients were excluded for the following reasons: a patient had the heart failure; 2 patients had the bacterial infection; a patient had the renal dysfunction. Some patients were classified as having advanced disease if they were not eligible for or had disease progression after surgical or locoregional therapies. Other patients, who were classified as having early disease, did not choose surgical or locoregional therapies. We used the Barcelona Clinic Liver Cancer (BCLC) Staging System for the determination of HCC stages.^(22)^ These patients were treated with antiemetic drugs; corticosteroid (dexamethasone), selective 5-HT_3_ antagonist (granisetron), and NK_1_ antagonist (aprepitant) to relieve the side effects of chemotherapy with either TACE or HAIC. The treatment with NK_1_ antagonist was identified as combination therapy of corticosteroid, selective 5-HT_3_ antagonist and NK_1_ antagonist. The treatment without NK_1_ antagonist was identified as combination therapy of corticosteroid and selective 5-HT_3_ antagonist.

### Study design

A retrospective study was performed to evaluate the risk factors of impaired liver function and to preventive effects of the impaired liver function for the antiemetic therapy with NK_1_ antagonist in cisplatin-naïve patients treated with TACE or HAIC using cisplatin. The Child-Pugh score is used as serum albumin, serum total bilirubin and prothrombin time for the assessment of the liver function. Because, some patients with HCC receive TACE or HAIC every 3 or 4 months, we measured the serum albumin, serum total bilirubin and prothrombin time by commercial methods prior to and 2 months after TACE or HAIC treatment using cisplatin. We used the minimum or maximum of the reference values as cut-off values for these parameters. The impaired liver function was identified as a change of Child-Pugh stage 2 months after TACE or HAIC which compared with prior. It is considered that one of the main roles assigned to albumin is as an indicator of the malnutrition.^(23)^ Therefore, the comparison of serum albumin between prior and after TACE was used for the deterioration of malnutrition in this study.

### Assessment of CINV

Same medical staffs maintained a diary for patients in which the timing and intensity of nausea and the timing and number of episodes of emesis were recorded. Subjects reported the severity of nausea and the number of emesis episodes from the day of treatment to the day for discharge. For assessing the severity of the nausea, vomiting, and anorexia, we utilized the Common Terminology Criteria for Adverse Events ver. 4.0 (CTCAE ver. 4.0). Acute CINV was identified as adverse events occurring within 24 h of initial administration of the chemotherapy. And delayed CINV was identified as occurring 24 h to several days after the initial chemotherapy.

### Statistical Analysis

Statistical analyses were performed using JMP Pro 10 software (SAS Institute Inc., Cary, NC). Differences in patient characteristics between patients with and without impaired liver function were compared using the Pearson’s chi-square test or the Fisher’s exact test for categorical variables and the Mann-Whitney *U* test for continuous variables. To determine the most suitable cut-off level of cisplatin’s dose, we used receiver operating characteristic (ROC) curve analysis. To identify risk factors of impaired liver function in patients treated with TACE or HAIC, multivariate analysis with a multiple logistic regression model were conducted including the variables with significant differences on univariate analysis. A *p* value of less than 0.05 was considered as a statistically significant.

## Results

### Comparison of clinical background of HCC patients after TACE or HAIC between with impaired liver function and without

**Fig. 1:**
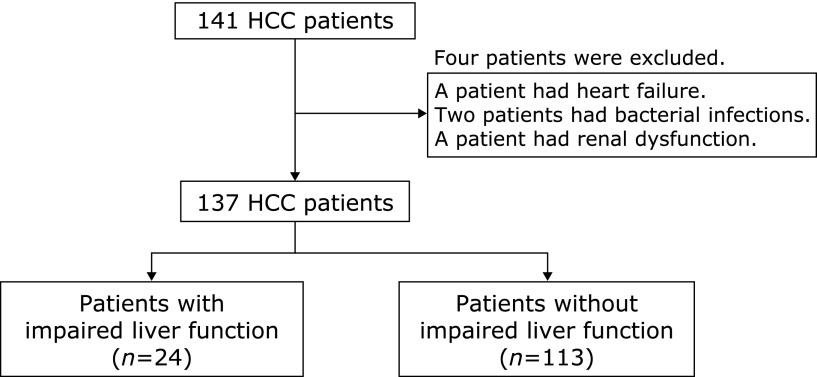
Flow chart of sample size.

A total of 137 cisplatin-naïve patients with HCC, who were received TACE or HAIC treatment using cisplatin, were enrolled in this study at Osaka Medical College. The impaired liver function was detected in 24 of these patients (Fig. 1). In comparing the clinical background of between these patients with impaired liver function or without, we found that there were no differences in age, gender, etiology, Child-Pugh score, performance status by Eastern Cooperative Oncology Group, history of angiography and embolization for HCC. Although some of the patients’ characteristics were known as risk factors for CINV (female, young and history of alcohol use), there were no differences between the two groups. The HCC stage by BCLC staging system was different between two groups and the impaired liver function in patients with intermediate or advanced stages of HCC were significantly more than in patients with very early or early stages. The dose of cisplatin was higher than the group with impaired liver function. Comparing for the treatment against CINV, liver dysfunction in the group of the treatment with NK_1_ antagonist was significantly less than without (Table 1). By univariate analysis of these patients, BCLC stage (intermediate or advanced), dose of cisplatin and treatment without NK_1_ antagonist were suggested as risk factors. As a result of multivariate analysis with a multiple logistic regression model, BCLC stage and treatment without NK_1_ antagonist were found to be independent risk factors of the impaired liver function in HCC patients after TACE or HAIC (Table 2). It is considered that liver function in patients with advanced stages of HCC will be deteriorated, regardless of the TACE or HAIC. Next, we studied the effect of treatment with NK_1_ antagonist against CINV on the liver function.

### The frequency of CINV in HCC patients after TACE or HAIC

**Fig. 2:**
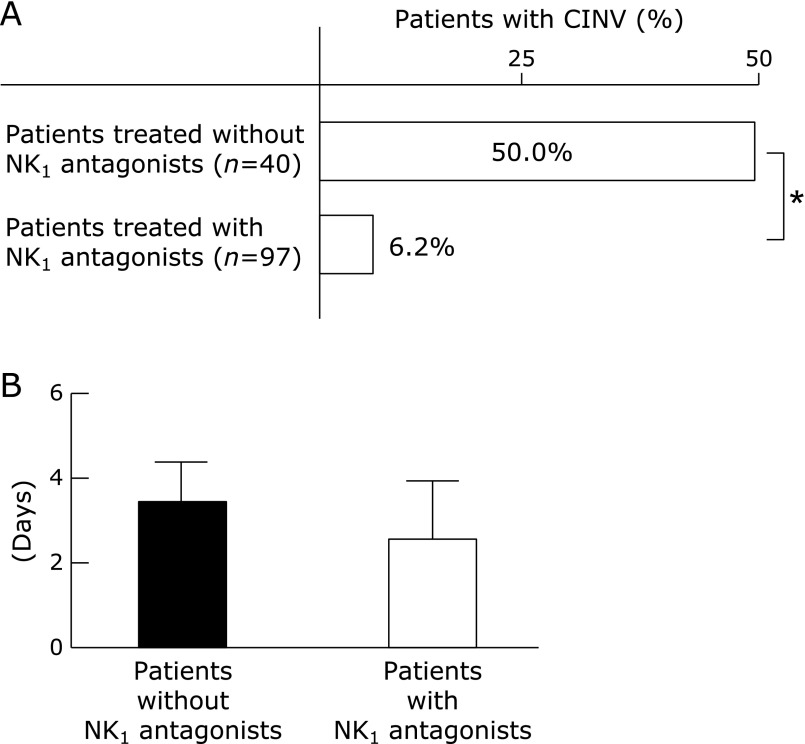
(A) Percentages of CINV in patients with NK_1_ antagonist or without after TACE or HAIC. The Pearson’s chi-square test was used to compare the percentages in each group. *p<0.05. (B) Average duration of CINV in HCC patients by treatment group. The Mann-Whitney *U* test was used to compare the average duration in each group.

We compared the incidence of CINV after TACE or HAIC between HCC patients treated with NK_1_ antagonist and without. Although, CINV was reported in 50% of patients treated without NK_1_ antagonist, only 6.2% of patients treated with NK_1_ antagonist were developed CINV after TACE or HAIC (Fig. 2A). We compared for the phases of CINV of these patients. Total 26 of patients had CINV in all patients and 11 of those patients had both CINV. A ratio of acute CINV was more than delayed CINV in patients without NK_1_ antagonist. On the contrary, a ratio of delayed CINV was more than acute CINV in patients with NK_1_ antagonist (Table 3A). Sixteen patients experienced delayed CINV with an average duration of 3.8 ± 1.7 days. There was no difference in an average duration between in patients with NK_1_ antagonist and without (Fig. 2B).

### The severity of CINV in HCC patients after TACE of HAIC

We compared the number and severity of CINV in patients with NK_1_ antagonist and without. The severity of adverse events in the patients was assessed by CTCAE ver. 4.0 and three adverse events (nausea, vomiting and anorexia) were reported in these patients. The prevalence of adverse events in both groups was shown in Table 3B. The number of patients with nausea was 20 and the number of anorexia was 22 in these patients. These adverse events were observed in both groups of with NK_1_ antagonist and without. However, 14 of vomiting were only reported in patients without NK_1_ antagonist. All of the CINV in these patients were grade 1 or 2 for CTCAE ver. 4.0 and no grade 3 CINV was observed.

The number and severity of adverse events in CINV patients were shown in Table 3C. Three adverse events were reported in the group of only acute CINV patients and in the group of both CINV. However, vomiting was not reported in the group of only delayed CINV patients. These results indicated that the treatment with NK_1_ antagonist was effective in reducing the frequency of both phases of CINV and vomiting in patients after TACE or HAIC.

### The preventive effect of the impaired liver function for treatment of NK_1_ antagonist

**Fig. 3:**
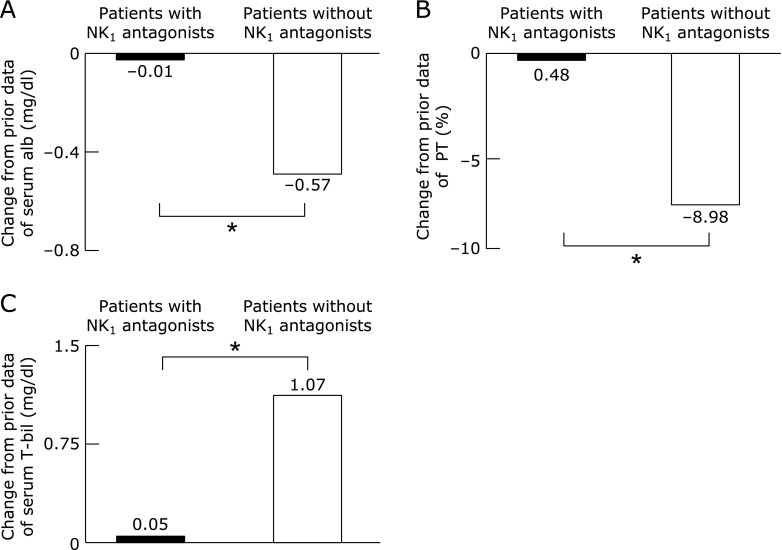
Change in serum albumin (A), prothrombin time (B) and serum total bilirubin (C) from prior to 2 months after TACE or HAIC in HCC patients. The Mann-Whitney *U* test was used to compare the data in patients with NK_1_ antagonist or without, respectively. *p<0.05.

The laboratory data which were evaluated in Child-Pugh score prior and 2 months after TACE or HAIC, were compared in patients with NK_1_ antagonist and without. Changes from prior data of serum albumin, prothrombin time and serum total bilirubin were shown in Fig. 3. Change in serum albumin from prior showed significantly reductions in patients without NK_1_ antagonist versus patients with NK_1_ antagonist. Because, serum albumin is the indicator of nutritional status, the malnutrition was deteriorated in patients without NK_1_ antagonist after TACE or HAIC. Furthermore, the change in prothrombin time from prior showed significantly reductions with patients without NK_1_ antagonist versus patients with NK_1_ antagonist and the change in serum total bilirubin was significantly increased on patients without NK_1_ antagonist. These data were little change in patients with NK_1_ antagonist. The deterioration of malnutrition which leads the impaired liver function, was not detected in patients with NK_1_ antagonist after TACE or HAIC. These results indicate that the treatment with NK_1_ antagonist may has a preventive effect on liver dysfunction caused by TACE or HAIC in HCC patients.

## Discussion

Cancer is one of the leading causes of death in the world, and its incidence has increased rapidly as the worldwide population continues to grow and age. Despite truly meaningful progress in targeted chemotherapy, many cancer treatment regimens still use cytotoxic chemotherapeutic agents that produce profound emesis and nausea that begin acutely on the day of chemotherapy and then reappear some days later.^(10,18)^ Patients beginning cancer treatment consistently list CINV as one of their greatest fears, and sometimes completely refuse or withdraw from therapy that has been prescribed to prolong or save their lives.^(24)^ And, CINV leads the deterioration of malnutrition in patients.^(10)^ Therefore, the control of CINV is a significant factor in ensuring patients’ quality of life during their treatment regiments to obtain the full benefit of chemotherapy.^(25)^ In this study, grade 1 and 2 adverse events were reported in our groups of HCC patients. However, grade 3 adverse event was not observed, whether patients did treat with NK_1_ antagonist or not. In TACE of HAIC, cisplatin was injected to the HCC selectively and it was retained in the tumor along with iodinated poppy seed oil. Because, that iodinated poppy oil works to keep the cisplatin in the tumor, cisplatin do not spread in whole body after TACE or HAIC. Therefore, the severe adverse events were reduced in these patients while increasing the antitumor effect.

The BCLC classification divides HCC patients in 5 stages according to the patient’s performance status, number and size of nodules, cancer symptoms, and liver function as determined by the Child-Pugh classification system.^(26)^ In this study, advanced stages of HCC (intermediate and advanced stage) was shown to be independent risk factors for the patients with impaired liver function after TACE or HAIC. BCLC staging system is one of a number of efforts to understand the prognosis for patients with HCC.^(6)^ It is also considered that liver function in patients with advanced stages of HCC will be deteriorated, regardless of the TACE or HAIC.^(3)^ Liver function was maintained with the treatment with NK_1_ antagonist during chemotherapy. Because the treatment with NK_1_ antagonist was very effective for CINV in these patients, the deterioration of malnutrition was not seen in patients after chemotherapy. The preventive effect of the treatment with NK_1_ antagonist retained liver function in HCC patients after TACE or HAIC.

For the treatment of HCC by TACE or HAIC, some antineoplastic agents are used in Japan. Epirubicin, commonly used for treatment of HCC in both TACE and HAIC since the 1990s, is categorized as having a moderate risk for CINV in guidelines for the prevention of CINV.^(27)^ In our hospital, the number of HCC patients who were treated with epirubicin by either TACE or HAIC was approximately fifteen per year, and the patients with CINV was less than those who were treated with cisplatin. In 2009 miriplatin, a new antineoplastic agent of platinum complexes was approved for the treatment of HCC by HAIC in Japan. This agent has a high affinity toward the iodinated poppy seed oil, and platinum components are released slowly from miriplatin suspended in iodinated poppy seed oil.^(28)^ Therefore, the total platinum concentration in the plasma shows a gradual change at a constant low concentration. There were few patients who were treated with miriplatin, and none had CINV in our hospital. Further study will be needed to assess this new drug.

NK_1_ receptor antagonist which developed as a treatment for CINV, acts by inhibiting the binding of substance P to the NK_1_ receptor in the vomiting center. Furthermore, it has reported that aprepitant is effective in the delayed phase of CINV.^(18,20,21)^ The treatment with NK_1_ antagonist in HCC patients treated with TACE or HAIC, was effective in both phases of CINV in our study. Nausea, anorexia, and vomiting were strongly suppressed by this therapy. This result is similar to that of aprepitant-containing antiemetic studies for other cancers.^(29,30)^ Although, the benefit of NK_1_ antagonist becomes apparent approximately 12 to 16 h after initiation of treatment, the therapeutic effect of NK_1_ antagonist was evident during both the acute and the delayed phases in this study.

In conclusion, BCLC stage and treatment without NK_1_ antagonist were found to be independent risk factors of the impaired liver function in HCC patients after TACE or HAIC. The treatment with NK_1_ antagonist was very effective in both phases of CINV associated with cisplatin-used TACE or HAIC in HCC patients.

## Figures and Tables

**Table 1:** Clinical background of these patients

	Patients with impaired liver function (*n* = 24)	Patients without impaired liver function (*n* = 113)	*p* value
Age (years)			
Mean (range)	70.9 ± 7.88	68.3 ± 9.97	0.293
Gender			0.624
Male	13	55	
Female	10	58	
Etiology			0.133
HBV	5	17	
HCV	18	75	
Others	1	21	
Child-Pugh Score	5.79 ± 0.93	5.81 ± 1.12	0.74
HCC stage (BCLC)			<0.001
Very early or early	2	59	
Intermediate or advanced	22	54	
Performance status			0.157
0	23	97	
1	1	15	
Alcohol drink/week (times)			0.858
0	19	87	
1–7	4	18	
>7	1	8	
History of angiography (+/–)	7/17	47/66	0.12
Serum albumin	3.41 ± 0.48	3.53 ± 0.50	0.209
Total bilirubin	1.16 ± 0.67	0.91 ± 0.47	0.106
Prothrombin time (%)	85.8 ± 12.0	89.0 ± 12.0	0.388
Dose of cisplatin (mg/body)	52.2 ± 27.6	34.6 ± 27.0	0.002
Emblization (+/–)	12/12	71/42	0.247
Treatment			0.004
with NK_1_ antagonists	10	87	
without NK_1_ antagonists	14	26	

**Table 2:** Hazard ratios of impaired liver function in patients after TACE or HAIC

	Hazard ratio	95%CI	*p* value
BCLC (intermediate or advanced)	12.7	3.32–84.29	<0.001
Dose of cislatin (mg/body)	1.01	0.99–1.03	0.372
Treatment without NK_1_ antagonist	3.36	1.14–10.23	0.002

**Table 3:** The severity and frequency of CINV in HCC patients after TACE or HAIC

(A) Number of CINV patients by treatment groups			
	Acute CINV	Delayed CINV	Both CINV
Patients without NK_1_ antagonist (*n* = 40)	18 (45.0%)	11 (27.5%)	9 (22.5%)
Patients with NK_1_ antagonist (*n* = 97)	3 (3.1%)	5 (5.1%)	2 (2.0%)

(B) Number and severity of adverse events in patients by treatment groups								
	Nausea (*n* = 20)			Vomiting (*n* = 14)			Anorexia (*n* = 22)	
	Grade 1	Grade 2		Grade 1	Grade 2		Grade 1	Grade 2
Patients without NK_1_ antagonists	10	6		11	3		9	7
Patients with NK_1_ antagonists	3	1		0	0		4	2

(C) Number and severity of adverse events in CINV patients								
	Nausea (*n* = 20)			Vomiting (*n* = 14)			Anorexia (*n* = 22)	
	Grade 1	Grade 2		Grade 1	Grade 2		Grade 1	Grade 2
Acute CINV only (*n* = 10)	5	2		6	2		4	3
Delayed CINV only (*n* = 5)	3	0		0	0		2	2
Both CINV (*n* = 11)	5	5		5	1		7	4

## Abbreviations

BCLC: Barcelona Clinic Liver Cancer
CINV: chemotherapy-induced nausea and vomiting
HACE: transarterial chemoembolization
HAIC: hepatic arterial infusion chemotherapy
HCC: hepatocellular carcinoma
